# A drug repositioning algorithm based on a deep autoencoder and adaptive fusion

**DOI:** 10.1186/s12859-021-04406-y

**Published:** 2021-10-30

**Authors:** Peng Chen, Tianjiazhi Bao, Xiaosheng Yu, Zhongtu Liu

**Affiliations:** College of Computer and Information Technology, China Three Gorges University, Hubei, China

**Keywords:** Drug repositioning, Adaptive fusion, Deep autoencoder

## Abstract

**Background:**

Drug repositioning has caught the attention of scholars at home and abroad due to its effective reduction of the development cost and time of new drugs. However, existing drug repositioning methods that are based on computational analysis are limited by sparse data and classic fusion methods; thus, we use autoencoders and adaptive fusion methods to calculate drug repositioning.

**Results:**

In this study, a drug repositioning algorithm based on a deep autoencoder and adaptive fusion was proposed to mitigate the problems of decreased precision and low-efficiency multisource data fusion caused by data sparseness. Specifically, a drug is repositioned by fusing drug-disease associations, drug target proteins, drug chemical structures and drug side effects. First, drug feature data integrated by drug target proteins and chemical structures were processed with dimension reduction via a deep autoencoder to characterize feature representations more densely and abstractly. Then, disease similarity was computed using drug-disease association data, while drug similarity was calculated with drug feature and drug-side effect data. Predictions of drug-disease associations were also calculated using a top-k neighbor method that is commonly used in predictive drug repositioning studies. Finally, a predicted matrix for drug-disease associations was acquired after fusing a wide variety of data via adaptive fusion. Based on experimental results, the proposed algorithm achieves a higher precision and recall rate than the DRCFFS, SLAMS and BADR algorithms with the same dataset.

**Conclusion:**

The proposed algorithm contributes to investigating the novel uses of drugs, as shown in a case study of Alzheimer's disease. Therefore, the proposed algorithm can provide an auxiliary effect for clinical trials of drug repositioning.

**Supplementary Information:**

The online version contains supplementary material available at 10.1186/s12859-021-04406-y.

## Background

Drug repositioning, also known as "conventional drug in new use", aims to search for new uses for drugs with existing indications. Conventionally, the development of new drugs is an expensive and inefficient process, requiring 10 to 15 years to launch a new drug on the market [1]. Targeted at the difficulty in conventionally developing new drugs, the computational analysis method of drug repositioning can offer a new way to develop new drugs, which has grown into a much-talked-about topic for researchers at home and abroad in recent years.

The research objective of drug repositioning was selected from the list of drugs approved by the US Food and Drug Administration (FDA). Thus, associated costs and risks can be decreased compared to studying unknown new drugs. Developing new drugs in a conventional way involves detection and clinical trials at early stages, safety review, clinical research, and post-marketing safety monitoring, while the drug repositioning method merely must go through compound identification, compound acquisition, drug development, and post-marketing FDA safety monitoring. In contrast, there has been an increasing number of successful cases of drug repositioning. For example, the original research goal of sildenafil was to treat vascular diseases, such as angina pectoris; however, its effect on treating male erectile dysfunction was unexpectedly discovered in the course of clinical testing [2]. Later, it was found from a follow-up study that low-dose sildenafil can also be used for treating rare pulmonary hypertension [3]. Colesevelam is a bile acid sequestrant with an initial indication of primary hypercholesterolemia and has been approved as a treatment drug for type-2 diabetes because its effect on controlling blood sugar was proven in an experimental study [4].

Certain occasional cases of drug repositioning can be found in earlier examples. However, a well-regulated and repeatable drug repositioning method has been studied by researchers at home and abroad after they discovered the significance of drug repositioning to the development of new drugs due to advancements in science and technology over time. Although the drug repositioning method that combines machine learning and deep learning has become mainstream, problems such as ineffective fusion of multisource data and sparse data remain an obstacle in drug repositioning. Cheng et al. [5] proposed and designed a complete, network-based drug repositioning method to determine new drug targets and anti-cancer indications by labeling significant mutant genes found in the human cancer genome. Zhang et al. [6] computed the drug-disease prediction upon measuring drug similarity with various data and inferred effective drug repositioning results via the fusion of all predictions with the maximum margin method interval method. In addition, the Area Under Curve (AUC) reached 0.8949. However, they failed to calculate the drug repositioning results from the disease-oriented perspective due to a single point of consideration. Zhang Jia et al. [7] computed the similarity of multiple data using the improved collaborative filtering equation and fused prediction results with an adaptive method. However, sparse data persisted. Luo et al. [8] proposed calculating the similarity of drugs and diseases using similar comprehensive methods of measurement; then, a network was constructed via the two similarities and fused into a heterogeneous network of drug-disease interactions. Then, a new drug-disease association could be predicted with two random walk models with AUCs reaching 0.917. However, multiple data sources cannot be rapidly and effectively fused using this method. Zeng et al. [9] developed a technique called deepDR to extract drug features from a heterogeneous network adopting multimodal autoencoders. Drugs that can be repositioned were inferred through encoding and decoding drugs with low dimensions using the variation autoencoder. In that case, the AUC reached 0.908, which was superior to the conventional network-based drug repositioning algorithm. In response to the global outbreak of 2019-nCoV, Zhou [10] proposed an antiviral drug reuse methodology and determined 135 types of drugs for the prevention and treatment of HCoV in accordance with a network medical platform based on system pharmacology.

DRDA, a drug repositioning algorithm that is based on deep autoencoder and adaptive fusion, is proposed in this paper to address the problems of data sparseness and low-efficiency fusion of multisource data in drug repositioning. In response to the sparseness of drug chemical structure and drug target protein data, two types of data are integrated into DRDA. The integrated data are known as "drug feature" data and allow more abstract features to be captured through dimension reduction in drug feature data via the deep autoencoder. After dimension reduction in features, disease similarity is computed with the drug-disease associated data, while drug similarity is computed with drug feature data and drug side-effect data. In addition, the predictions of drug-disease associations are calculated using a top-k neighbor that is commonly used in predictive drug repositioning studies. Finally, the weight of various data sources is adjusted via adaptive fusion to decrease the weight of data sources that cannot provide effective information for drug repositioning. Experimental results show that the designed drug repositioning algorithm can effectively reduce data sparseness and adjust the weight of a data source to improve the precision and recall rate. The primary contributions of this study are as follows:

1. A general drug repositioning framework fusing information from multiple data sources was developed to effectively perform drug repositioning computations.

2. A deep autoencoder was designed to perform dimension reduction on drug feature data (drug chemical structure and drug target protein), which can extract abstract features. Thus, the autoencoder can mitigate data sparseness and improve computation efficiency.

3. A weight computation method that is more appropriate for drug repositioning was designed for multisource data fusion, guaranteeing the effect of algorithm indicators and enhancing the prediction ability of new uses of drugs.

4. Experimental results show that DRDA achieves excellent performance in all indicators (precision, recall rate and F-score). Precision reaches 0.9047 with an F-score of 0.9041, yielding a better prediction result in drug-disease association due to the adaptive fusion method.

The remainder of this paper is structured as follows. “Methods” section introduces the basic concepts of an encoder. In addition, DRDA is proposed in “Results” section. Next, DRDA is analyzed and verified via various sets of experiments in “Discussion” section, where related cases are shown. In “Conclusion” section, the work that has been conducted and future research directions are concluded.

## Methods

**Fig. 1:**
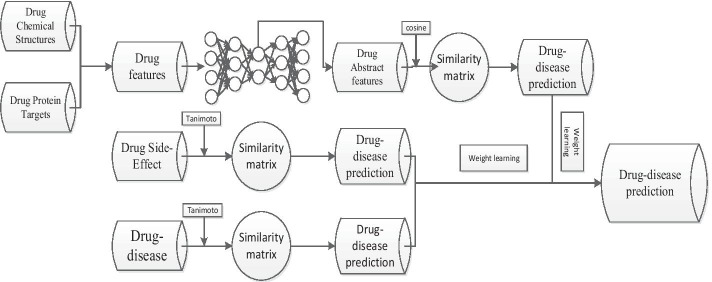
Framework of the drug repositioning algorithm based on a deep autoencoder and adaptive fusion, including four types of origin data and three types of data for experiments

DRDA, a drug repositioning algorithm that is based on a deep autoencoder and adaptive fusion, is proposed in this paper to address the problems of data sparseness and low-efficiency fusion of multisource data in drug repositioning. First, dimension reduction was performed on drug features, including drug chemical structures and drug target proteins, using a deep autoencoder before extracting more abstract representations of drug features. Then, drug similarity was computed using drug features and drug side-effect data, and disease similarity was computed using drug-disease associated data. The prediction of drug-disease association was computed using the top-k similar neighbor method, which is more suitable for drug repositioning. Finally, the prediction of the drug-disease association was determined with the fusion of predictions computed by various data sources utilizing adaptive fusion. The algorithm framework is shown in Fig. 1.

### Dimension reduction in drug features based on the deep autoencoder

**Fig. 2:**
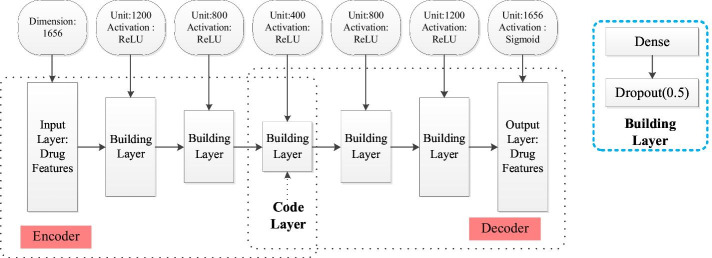
A schematic diagram of a deep autoencoder framework for drug feature data dimensionality reduction. There are two sections in the overall structure: the encoder, and the decoder. The encoder contains four layers: an input layer, two building layers and an encoding layer. The decoder includes four layers: an encoding layer, two building layers, and an output layer. The building layer is composed of a dense and dropout layer

To reduce the sparseness of drug chemical structure and drug target protein data, two types of data were integrated into drug feature data. Then, more abstract drug features were extracted to decrease data sparseness by reducing the dimensions of drug features with a deep autoencoder. The deep autoencoder is an extension of the autoencoder, which converts high-dimensional data to low-dimensional data via a multilayer encoding network and recovers the encoding with a similar decoder. A training network for errors between input data can be reconstructed by minimizing the original data [11]. Thus, a deep autoencoder that is more relevant to drug feature data was designed. Its structure is shown in Fig. 2.

The deep autoencoder is composed of an encoder and a decoder. "Adagrad" [12] is regarded as an optimization method, and the encoder consists of an input layer and three building layers [13]. Specifically, the building layer is composed of a fully connected layer and a discarded layer, and the last building layer is a coding layer. All layers contain a set of fully connected layers and a discard layer with a parameter set to 0.5. In addition, ReLU is used as the activation function. When dimension reduction is performed on drug feature data with drug feature data $x \in R^{m \times n}$ as input, the building layer is computed in Eq. (1):

1 $$g^{i} (x) = f(\omega^{i} g^{*} (x) + b^{i} )$$

where $g^{i} (x)$ is the output of the building layer; $f( \cdot )$ is a nonlinear activation function; $\omega^{i} ,b^{i}$ represents the weight and offset of the ith building layer, $$g*(x) = \left\{ {\begin{array}{*{20}c} x & {i = 1} \\ {g^{i - 1} (x)} & {i > 1} \\ \end{array} } \right.$$; and i is the number of building layers.

**Fig. 3:**
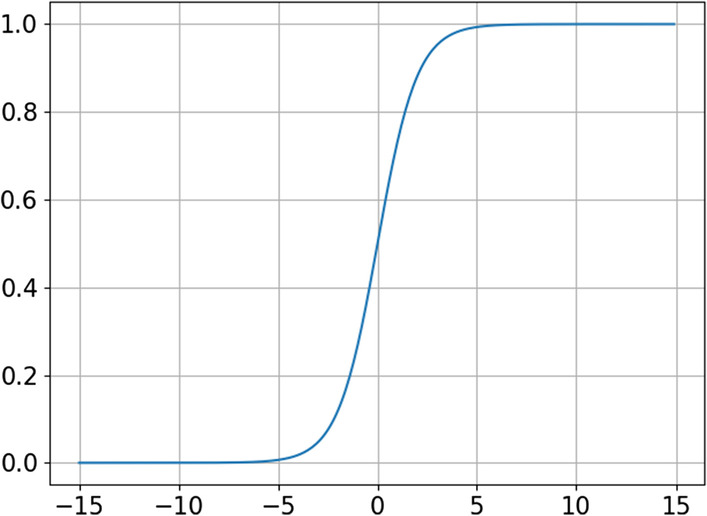
Sigmoid function image

The decoder consists of three building layers and an output layer; the first building layer is an encoding layer. ReLu is used as the activation function in all layers except the output layer, which uses the sigmoid function as its activation function. Because drug feature data are binary, the reconstructed output should be data approaching 0 or 1 to reduce errors between the reconstructed output and input as quickly as possible. Most of the output values of the sigmoid function are concentrated at approximately 0 and 1 (Fig. 3), which follows the structure required by the output. The computation method of the decoder building layer is similar to that of the encoder building layer. The decoder output layer can be computed in Eq. (2):

2 $$\mathop x\limits^{\_} = g^{4} (x) = f(\omega^{3} g^{3} (x) + b^{3} )$$

The mean square error is applied in the deep autoencoder as the loss function. The gap between the input *x* and the output $\mathop x\limits^{\_}$ upon reconstructed input can be decreased by minimizing the mean square error. With the minimum error, extracting the features of the encoding layer is the feature data after dimension reduction.

The training parameter batch size used is 16, and the learning rate of the optimization function "Adagrad" is 0.01. Before training, parameters are initialized via Xavier [14]. In addition, 400-dimension feature data extracted from the output of the encoding layer are used for subsequent computation of drug similarity.

### Similarity computation

#### Drug similarity

Dudley [15] and Li [16] et al. stated that drug chemical structure and drug target proteins play a critical role in calculating drug similarity because they are quantitatively related. Drugs with similar target proteins can also treat similar diseases. Drug chemical structure data and drug target protein data were sampled from PubChem [17] and the UniPort Knowledgebase [18]. After dimension reduction of drug feature data with the aforementioned deep autoencoder, the drug features that are denser than the original data can be obtained. Then, cosine similarity is used to calculate drug similarity, as shown in Eq. (3):

3 $$sim(d,d*) = \frac{{\sum\limits_{i = 1}^{n} {{\text{f}}_{{d_{i} }} \cdot {\text{f}}_{{d_{i}^{*} }} } }}{{\sqrt {\sum\limits_{i = 1}^{n} {{\text{f}}_{{d_{i} }}^{2} } } \cdot \sqrt {\sum\limits_{i = 1}^{n} {{\text{f}}_{{d_{i}^{*} }}^{2} } } }}$$

where $sim(d,d*)$ represents the similarity of the drug $d$ and the drug $d*$; ${\text{f}}_{{d_{i} }}$ and ${\text{f}}_{{d_{i}^{*} }}$ stand for the value of the ith drug feature in the drug $d$ and the drug $d*$, respectively; and *n* is feature dimension.

Verifying whether two drugs can act on the same target through drug side effect data was proposed in the literature [19]. Additionally, a series of experiments were designed to demonstrate the feasibility of inferring molecular interactions using side effect data. Thus, the drug side effect data can be used to calculate drug similarity. Drug side effect data were sampled from the SIDER [20] database. If the drug causes a specific type of side effect, the value is set to 1; if not, it is set to 0. The Tanimoto coefficient is used for computation in Eq. (4):

4 $$sim(d,d*) = \frac{{{|}I_{dd*} {|}}}{{|I_{d} | + |I_{{d^{*} }} | - |I_{{dd^{*} }} |}}$$

where $I_{dd*}$ represents the number of the same side effects of the two drugs, and $I_{d}$ and $I_{{d^{*} }}$ represent the number of side effects of the two drugs, respectively.

#### Disease similarity

In the literature [21], an inferred idea associated with drug repositioning has been proposed: two diseases are deemed similar when they can be treated by a variety of identical drugs. According to the method proposed in the literature [7], disease similarity can be computed with drug-disease associated data sampled from UMLS [22]. The data are binary: if the drug has a treatment effect on the disease, it is set to 1; otherwise, it is set to 0. When the Tanimoto coefficient is used for computation, Eq. (5) can be used:

5 $$sim(e,e*) = \frac{{{|}I_{ee*} {|}}}{{|I_{e} | + |I_{{e^{*} }} | - |I_{{ee^{*} }} |}}$$

where $sim(e,e*)$ is the similarity of the two diseases; $I_{ee*}$ is the number of drugs that can treat the two diseases; and $I_{e}$ and $I_{{e^{*} }}$ represent the number of drugs that can treat the diseases *e* and e*, respectively.

### Computation of prediction

Only "0" and "1" relationships between the drug and disease can be found in the original drug-disease associated data, while certain side-effect relationships may be detected between the drug and some diseases. To calculate the drug-disease associated prediction effectively, the known drug-side effect relationship in the drug-side effect data was marked in the drug-disease data: if a side effect (disease) exists in both the drug-disease associated data and drug-side effect data, the corresponding drug-side effect (disease) should be changed from "0" to "−1" in the drug-disease associated data when the drug produces the side effect.

As shown in the literature [7, 23], the prediction of drug-disease association in drug repositioning can be computed using collaborative filtering. However, the drug-disease prediction of the conventional approaches of collaborative filtering and top-k neighbor cannot be accurately computed because data used in drug repositioning are typically sparse, and there is a side effect relationship between drugs and diseases. In this paper, the drugs or diseases to be evaluated were also computed as similar neighbors based on top-k proximity. With a small number of effective neighbors (i.e., the similarity is not 0) caused by data sparseness, the drug-disease associated information of the drug or disease is decisive, which can avoid false predictions resulting from a lack of effective neighbors. When there are sufficient effective neighbors, the drug-disease associated information of the drug or disease is only one of the neighbors, exerting a small effect on predicting the new effect of the drug. Fusing known information with the prediction can lower the prediction error caused by a lack of effective neighbors due to data sparseness. Applying known information about drugs or diseases for computation can avoid computational collapse resulting from an effective neighbor being zero.

Drug-disease associated prediction can be computed through drug similarity, as shown in Eq. (6):

6 $$P_{de}^{k} = \frac{{\sum\limits_{{d^{*} \in {\text{NN}}^{\prime}}} {sim^{k} (d,d^{*} ) \times s_{{d^{*} e}} } }}{{\sum\limits_{{d^{*} \in {\text{NN}}^{\prime}}} {sim^{k} (d,d^{*} )} }}$$

where $P_{de}^{k}$ is the predicted score between drug *d* and disease *e* computed based on data source *k*; ${\text{NN}}^{\prime}$ is the union set of drug $d$ and its top-k neighbors; and $s_{{d^{*} e}}$ is the relational value between the drug $d^{*}$ and the disease e upon integrating drug-side effect data in the drug-disease associated dataset.

The computation of the drug-disease associated prediction for disease similarity is similar to that of drug similarity, as shown in Eq. (7):

7 $$P_{de}^{k} = \frac{{\sum\limits_{{e^{*} \in {\text{NN}}^{\prime}}} {sim^{k} (e,e^{*} ) \times s_{de*} } }}{{\sum\limits_{{e^{*} \in {\text{NN}}^{\prime}}} {sim^{k} (e,e^{*} )} }}$$

where ${{\text{NN}}^{\prime}}$ is the union set of disease *e* and its top-k neighbors.

The drug-disease associated prediction that finally fuses multiple data sources is calculated as Eq. (8):

8 $$P_{de}^{*} = \sum\limits_{k = 1}^{{\text{K}}} {\beta_{k} \times P_{de}^{k} }$$

where $P_{de}^{*}$ is the prediction of the drug *d* after data fusion to the disease *e*; $\beta_{k}$ is the weight of the k data source; and $P_{de}^{k}$ is the prediction of the drug *d* to the disease *e* in the k data source.

### Weight computation

A weight computation method that is more suitable for drug repositioning was designed per the literature [7] to fuse the predictions computed by multiple data sources. In addition, the best prediction effect was achieved by maximizing the combination of the drug-disease associated value of 0 and the difference between its predictions while minimizing the difference between the combination of the drug-disease value of 1 and its prediction. The weight computation method can be expressed as an optimization objective function, such as (9):

9 $$\begin{aligned} \mathop {\arg \min }\limits_{{\beta_{k} }} L(\beta_{k} ) & = \sum\limits_{k = 1}^{k} {\beta_{k}^{2} } \left( {\sum\nolimits_{{\{ (d,e)|s_{de} = 1\} }} {\left( {s_{{d{\text{e}}}} - p_{de}^{k} } \right)^{2} - } \sum\nolimits_{{\{ (d,e)|s_{de} = 0\} }} {\left( {s_{de} - p_{de}^{k} } \right)}^{2} } \right) \\ {\text{s.t}}.\,\sum\limits_{k = 1}^{K} {\beta_{k} } & = 1 \\ {\text{s.t.}}\,\beta_{k} & > 0 \\ \end{aligned}$$

where $\{ (d,e)|s_{de} = 1\}$ represents the combination of the associated value being 1 between drug *d* and disease *e* in the drug-disease associated data. Similarly, $\{ (d,e)|s_{de} = 0\}$ is the drug-disease combination being 0. This formula is intended to increase the weight of data sources that can make the predicted value as large as possible. $\sum\nolimits_{{\{ (d,e)|s_{de} = 1\} }} {(s_{{d{\text{e}}}} - p_{de}^{k} )^{2} }$ indicates that when the drug *d* can treat the disease *e* in the known data, the predicted value and drug must be reduced as much as possible. The disease-related value thus keeps the predicted value near "1". Similarly, ${ - }\sum\nolimits_{{\{ (d,e)|s_{de} = 0\} }} {(s_{de} - p_{de}^{k} )}^{2}$ indicates that when drug *d* cannot treat disease *e* in the known data, it is necessary to make the prediction value as large as possible because the repositioning of the drug is determined by the concept that the new use of the drug is predicted. This process is necessary to predict drug-disease combinations with known relationships and to develop a more reasonable and effective unknown drug-disease combination. The optimized problem is solved using the minimization method in scipy of the Python package.

### Algorithm flow

The overall algorithm flow is presented as follows:
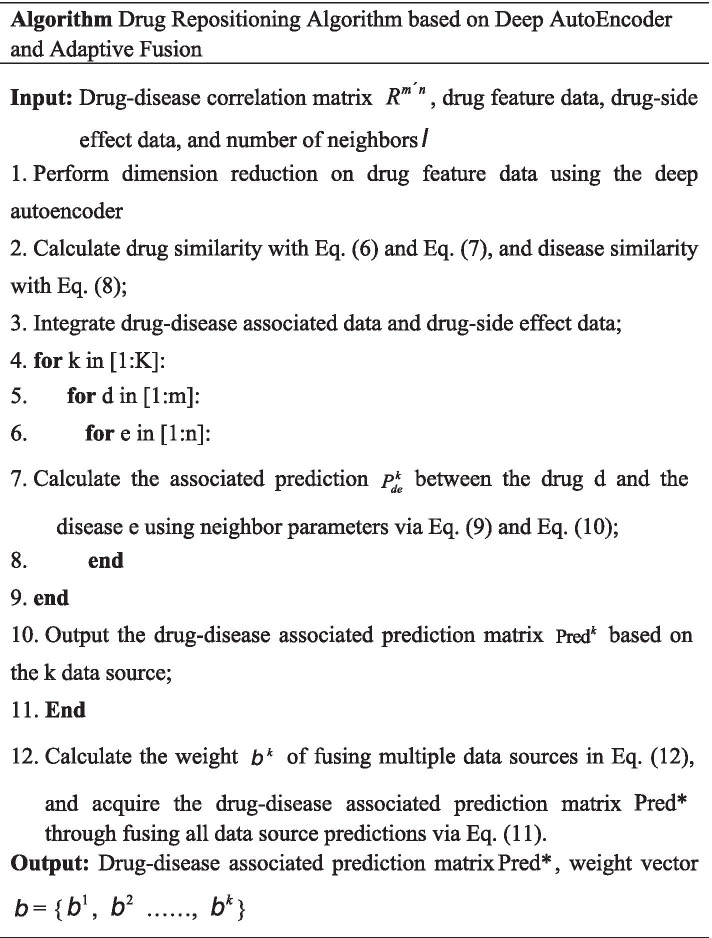


## Results

To demonstrate the effectiveness and feasibility of the proposed algorithm, data source comparison, method comparison and case analysis are explained experimentally.

### Dataset

**Fig. 4:**
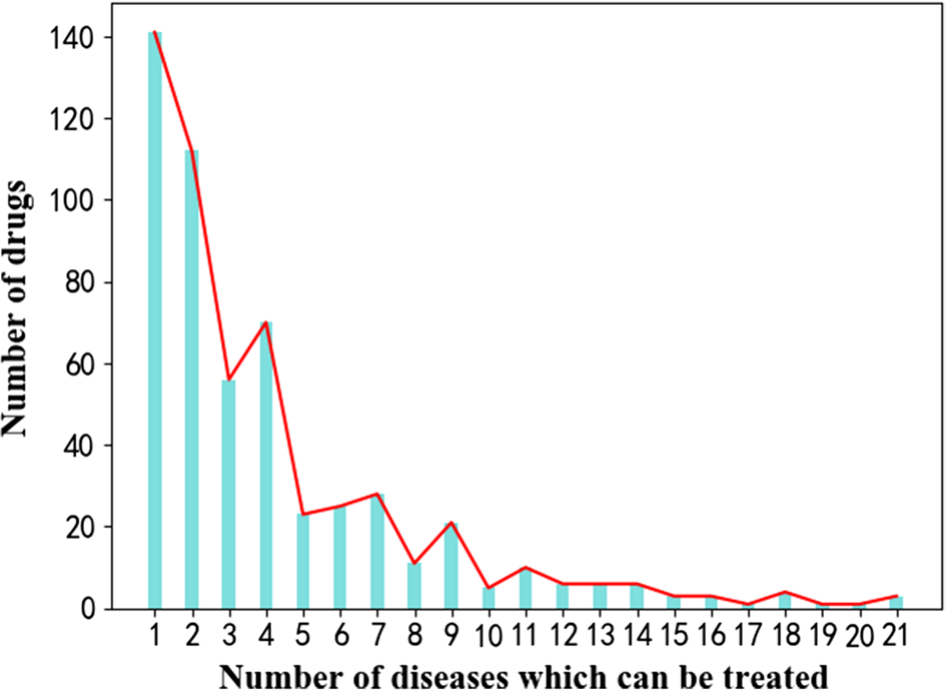
The number of diseases that can be treated by each drug in the drug-disease associated data

The dataset used in the experiment contains a drug disease-associated dataset, a drug feature dataset (drug target proteins and drug chemical structures), and a drug side-effect dataset. Specifically, the drug disease-associated dataset covers 536 drugs and 578 diseases, including 2229 drugs with known treatment effect-disease association; the drug feature dataset includes 775 target proteins and 881 chemical structures; and the drug side-effect dataset contain 1385 side effects. The sparse degrees of the three datasets (the proportion of invalid data in data) are 0.9928, 0.9231, and 0.9455, respectively. Dimension reduction cannot be performed on the drug-disease associated and drug-side effect data due to their particularity. However, dimension reduction was performed on the drug features data with the deep encoder to extract more abstract expressions. The sparse degree of the drug feature dataset upon dimension reduction is 0.7703 (a 16% reduction). The number of diseases that can be treated by each drug in the drug-disease associated data are shown in Fig. 4. Among these, the classification of drugs is mutually exclusive; thus, drugs for the treatment of two diseases are not included in the category of drugs for the treatment of one disease. Therefore, most drugs can only treat fewer than five types of diseases; thus, drug-disease association data are particularly sparse.

### Experimental indicators

The drug repositioning task in the experiment can be deemed a binary classification task [6]. For a certain disease, the corresponding relationship is 1 if the drug can treat it and 0 if it cannot. The precision, recall rate, F-score and ROC curve are experimental indicators. First, a confusion matrix is defined in Table 1 before defining the above four indicators. TP is the result of a drug-disease correlation value of 1 and a prediction of 1. FN is the result of a drug-disease correlation value of 1 and a prediction of 0. FP is the result with an original value of 0 and a predicted value of 1. TN is the result where the original value is 0 and the predicted value is 0. Then, the precision and the recall rate are defined as Eq. (10) and Eq. (11):

10 $$P = TP/(TP + FP)$$

11 $$R = TP/(TP + FN)$$

**Table 1:** Confusion matrix

	Prediction	
True value	TP (True positive)	FN (False negative)
	FP(False positive)	TN (True negative)

Then, the F-score can be computed, as shown in Eq. (12):

12 $$F = 2 \times P \times R/(P + R)$$

In addition, the AUC is used as the experimental indicator. Because a specific threshold is required to divide the predicted score into 1 and 0, the threshold should be set in a way that can achieve the best effect of the F-score. The overall experiment was performed with tenfold cross-validation.

### Comparison of data sources

**Fig. 5:**
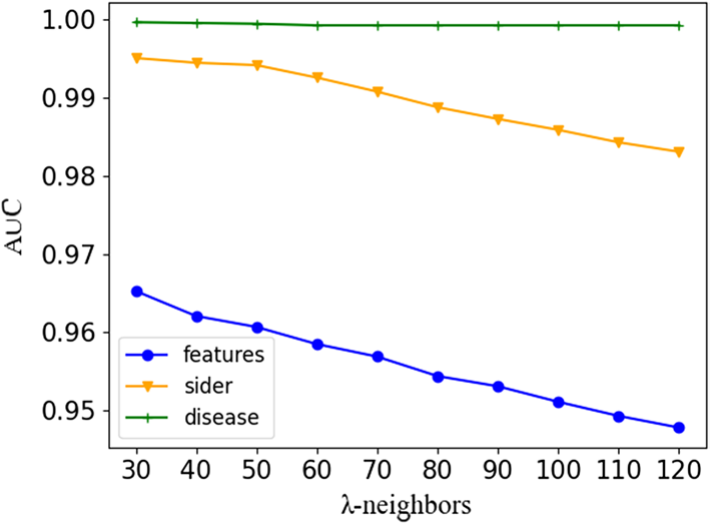
Changes in the AUCs of the three data sources with different numbers of neighbors (30–120). Via comparison, we find that although the AUC values of three data sources are the highest under 30 neighbors, drug repositioning is a prediction task rather than a simple classification task, and we need more neighbors to provide information. Thus, we choose 50 as the number of neighbors

To determine the number of neighbors that can achieve the best algorithm effect, changes in the AUCs of the three types of data under different numbers of neighbors are given. In addition, computation results with over 50 neighbors are given to ensure that there are sufficient neighbors to provide information, as shown in Fig. 5. The observation chart shows that the AUC of the disease association dataset does not change markedly with the change in the number of neighbors, while the sider data have a relatively large decline after 50 neighbors, and the feature data decrease with the increase in neighbors. However, DRDA is an algorithm that is designed to predict new uses of existing drugs. We should perform experiments on the premise of sufficient neighbors because more neighbors can provide more new predictions. If only a few drugs are similar, the results are closer to the classification rather than the prediction of unknown new drug disease associations. Therefore, 50 is selected as the number of similar neighbors.

After determining the values of the similar neighbor parameters $\lambda$, weight computation is performed in accordance with the method mentioned in Sect. 3.4. The highest weight of the drug-disease associated data source is 0.6605 among the three data sources, and the drug feature data and drug side effect data account for 0.1356 and 0.2037, respectively. The three data sources are computed separately and compared with DRDA in terms of precision, recall rate, F-score and AUC values, as shown in Table 2. Four indicators of the drug-disease associated data are the highest among the three data sources, with a precision rate of 0.9823 and an AUC value of 0.9998. The recall rate of drug features can reach up to 0.8593, while the precision rate is extremely low, reaching 0.2082. Similarly, the drug-side effect can reach a recall rate of 0.6008, yet its precision is only 0.3090. Apparently, the prediction computed by a single data source is unstable and cannot complete the task of drug repositioning well. DRDA fuses the results of the three data sources through an adaptive method, which can obtain results that are superior to the three data sources, achieving a precision of 0.9047 and an F-score of 0.9041.

**Table 2:** Comparison of three data sources and three indicators of DRDA

Datasets	Precision	Recall	F-score	AUC
Drug-disease	0.9823	0.7167	0.8287	0.9998
Drug feature	0.2082	0.8593	0.3351	0.9649
Drug-side	0.3090	0.6008	0.4081	0.9940
DRDA	0.9047	0.9035	0.9041	0.9993

As shown by the AUC indicator in Table 2, high AUCs with all data sources do not imply that the results of all data sources conform to ideal values because drug repositioning is an extremely unbalanced problem. In the drug-disease associated data, the number of drug-disease associations being 1 is far less than the number of drug-disease associations being 0. Consequently, the AUCs are blindly optimistic [24].

### Method comparison

**Fig. 6:**
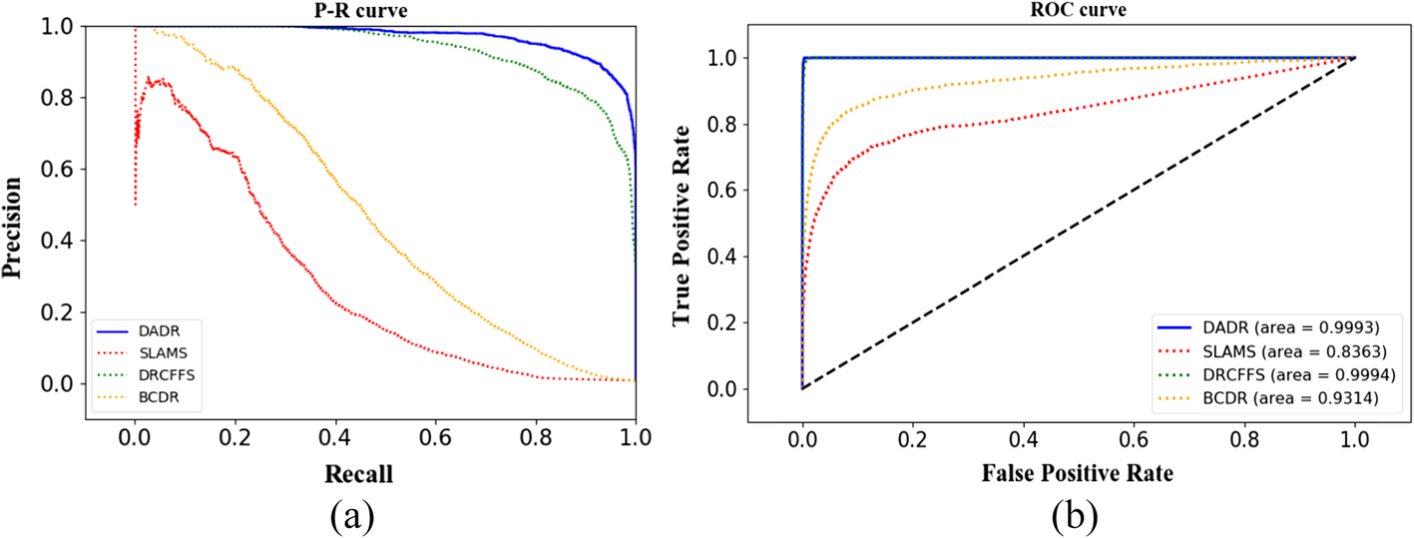
**a** PR curves of the DRDA algorithm and the other three algorithms. The PR curve of DRDA wraps the PR curves of the other three algorithms; thus, the effect is the best. **b** ROC of the four algorithms. Comparing the curve and AUC value, DADR and DRCFFS have the best effect

To evaluate the performance of DRDA, DRDA was compared with SLAMS [6], DRCFFS [7], and DRBC proposed in the literature [23]. The three algorithms were tested for tenfold cross validation, and the average value was used to ensure rationality and fairness. In the experiment, 50 is the most suitable neighbor number for DRDA. However, in other experiments, the other two algorithms do not yield a specific number of neighbors except DRCFFS, which explicitly outputs 90 neighbors. Therefore, the numbers of neighbors of the SLAMS, DRCFFS, and DRBC algorithms are all set equal to 90. As shown in Fig. 6a, the P–R curve of DRDA wraps the P–R curves of the other three algorithms. The ROC curves of the four algorithms are shown in Fig. 6b. The AUC values of DRDA and DRCFFS are similar and the highest among the four algorithms, being 0.9993 and 0.9994, respectively. The AUC of SLAMS was the lowest (0.8363). Also, the precision, recall rate and F-score of the three algorithms and DRDA were also compared in the experiment, as shown in Table 3. DRDA achieved superior performances with the three indications compared to the other three conventional algorithms, reaching a precision rate of 0.9047 and a recall rate of 0.9035. Compared with DRCFFS, the best existing drug repositioning algorithm, DRDA achieves better performances on the other three indicators than DRCFFS, although DRDA has a marginally lower in AUC. In particular, its recall rate is 0.1104 higher than that of DRCFFS‬. Additionally, DRDA pays more attention to the prediction of unknown drug-disease associations under the premise of guaranteeing indicators. Overall, DRDA achieves better performance than the conventional drug repositioning algorithm.

**Table 3:** Comparison of precision, recall rate and F-score indicators of the four algorithms with 90 neighbors

Algorithm	Precision	Recall	F-score	AUC
SLAMS	0.1494	0.4984	0.2299	0.8363
DRCFFS	0.8778	0.7931	0.8333	0.9994
DRBC	0.7367	0.2768	0.4024	0.9314
DRDA	0.9047	0.9035	0.9041	0.9993

Concurrently, the experimental results of DRDA, SLAMS, DRCFFS, and DRBC with 50 neighbors are compared, as shown in Table 4. Similar to the experiment with 90 neighbors, the performance of DRDA in four indicators is found to be better than the other three algorithms.

**Table 4:** Comparison of precision, recall rate and F-score indicators of the four algorithms with 50 neighbors

Algorithm	Precision	Recall	F-score	AUC
SLAMS	0.1902	0.5488	0.2825	0.8527
DRCFFS	0.8529	0.8375	0.8452	0.9993
DRBC	0.7582	0.3397	0.4692	0.9398
DRDA	0.9047	0.9035	0.9041	0.9993

**Fig. 7:**
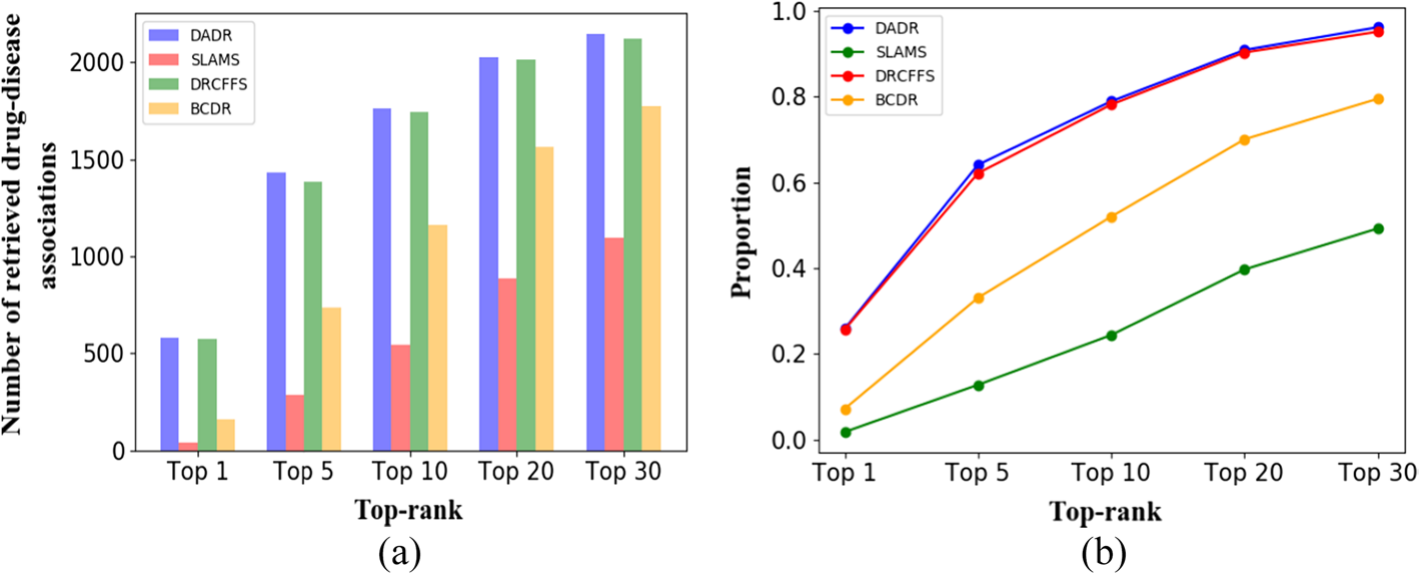
**a** Correct prediction of the drug-disease associated number with known treatment relationships for the four algorithms at different thresholds. **b** Proportion of the number of drug-disease associations correctly predicted by the four algorithms with known treatment relationships at different thresholds to all drug-disease with known treatment relationships

The drug-disease associated numbers (the associated value of the drug and the disease is 1) correctly predicted by the four algorithms are given at different thresholds to extensively compare the drug-disease associated prediction effects of DRDA, DRCFFS, SLAMS, and DRBC. Figure 7a shows that the abilities of DRDA and DRCFFS to predict drug-disease associations with known treatment effects are better than those of the other two algorithms. As the threshold is gradually relaxed, DRDA yields better performance than DRCFFS. The proportions of the total number of drug-disease associations correctly predicted by the four algorithms with known treatment relationships at different thresholds to all drug-disease with known treatment relationships are shown in Fig. 7b. DRDA and DRCFFS nearly predicted all drug-disease combinations with known treatment relationships in the course of selecting drugs with the top 30 predicted scores of each disease. In addition, unlike DRCFFS, DRDA also pays attention to the prediction of unknown drug-disease associations. Thus, the predicted score of the drug-disease associated value being 0 in the original dataset is maximized in Eq. (12), apart from predicting the drug-disease association with known treatment relationships. Hence, DRDA can predict drug-disease associations with unknown existing relationships on the premise of ensuring the effective prediction of the number of drug-disease associations with known treatment relationships.

Table 5 shows that DRDA is marginally lower than the simple average fusion method over the precision, while DRDA is 0.2364 higher than the simple average fusion method over the recall rate. Thus, the simple average fusion method cannot effectively filter the data added subsequently, which is not conducive to the fusion of multisource data. DRDA is thus shown to yield superior performance compared to the simple average fusion method in theory and indicators.

**Table 5:** Comparison of DRDA and the simple average fusion method over precision, recall rate and F-score

Algorithm	Precision	Recall	F-score
DRDA	0.9047	0.9035	0.9041
Simple Avg	0.9441	0.6671	0.7818

### Case study

To demonstrate that DRDA can effectively assist drug repositioning, the drugs of the top ten predictions for Alzheimer's disease (AD) and their original uses are shown in Table 6. Four drugs have been used for the clinical treatment of Alzheimer's disease, and five out of the remaining six drugs have been studied for the treatment of Alzheimer's disease. However, this does not imply that all drug repositioning results predicted by DRDA are feasible because the development of drugs is a long and complex process. Apparently, the case analysis showed that DRDA could save time for developing new drugs by providing theoretical and data support for drug repositioning and assistance in the research direction.

**Table 6:** Drugs with the top 10 scores for treating Alzheimer's disease

Drug Name	Origin Use	Study on the treatment of AD
Clozapine	Schizophrenia	[26, 27]
Pramipexole	Parkinsonism	[28]
Olanzapine	Schizophrenia	[29]
Carbamazepine	Epilepsy Peripheral neuralgia	[30]
Donepezil	AD	Used in clinical treatment of Alzheimer's disease
Ethosuximide	Clonic	No research
Galantamine	TO	Used in clinical treatment of Alzheimer's disease
Rivastigmine	AD	Used in clinical treatment of Alzheimer's disease
Selegiline	AD	Used in clinical treatment of Alzheimer's disease
Valproic Acid	Epilepsy	[31]

Concurrently, to demonstrate that the case analysis of AD is not an accidental event, we report the drugs with the top five predictive scores of atrial fibrillation and dyspepsia, as shown in Tables 7 and 8. The drugs with the top five predictive scores in the predicted drug atrial fibrillation association combination have been used in the clinical treatment of atrial fibrillation. Only cimetidine has been used in the clinical treatment of dyspepsia, and amoxicillin combined with other drugs has been studied in the treatment of dyspepsia. The top two drugs among the other three drugs include bacitracin and thiophosphate, which are used in the treatment of gram-positive bacteria and glaucoma, respectively. There is no research on these two drugs, which are associated with the treatment of dyspepsia.

**Table 7:** Drugs with the top 5 scores for treating atrial fibrillation

Drug Name	Origin Use	Study on the treatment of AF
Propipramine	AF	Used in clinical treatment of AF
Diltiazem	AF	Used in clinical treatment of AF
Digoxin	AF	Used in clinical treatment of AF
Atenolol	AF	Used in clinical treatment of AF
Dofett	AF	Used in clinical treatment of AF

**Table 8:** Drugs with top 5 scores for treating Dyspepsia

Drug Name	Origin Use	Study on the treatment of Dyspepsia
Bacitracin	Gram positive bacteria	No research
Thiophosphate	Glaucoma	No research
Cimetidine	Dyspepsia	Dyspepsia
Amoxicillin	Bacterial Infection	[31]
Enflurane	–	No research

## Discussion

In this section, we verify the performance of the DRDA algorithm through experiments and a case study, and the results demonstrate the effectiveness and feasibility of DRDA. A case study shows that most of the drugs predicted by the DRDA algorithm to treat Alzheimer's disease have been studied.

Concurrently, we also performed a case analysis of the other two drugs and found that the DADR algorithm proposed in this paper still has some problems in the prediction of some drug associations. For example, in the prediction of drugs for the treatment of dyspepsia, only one drug in the top five is in this study.

Finally, we believe that the experimental form of binary classification is disadvantageous to the drug repositioning algorithm because the drug repositioning algorithm primarily predicts unknown associations, while the binary classification method is based on the existing data for comparison and experiment, which does not conform to the idea of predicting unknown associations.

## Conclusion

A drug repositioning algorithm based on a deep autoencoder and adaptive fusion (DRDA) was proposed in this study (Additional file 1). Specifically, dimension reduction was performed on data via the deep autoencoder to decrease the impact resulting from data sparseness. The weights of all data sources were also computed to fuse the information of various data sources. Experimental results show that DRDA yields superior performance compared to the conventional drug repositioning algorithm, reaching a precision of 0.9047. In addition, a weight computation method that is more suitable for drug repositioning was also designed to ensure the quality of indicators and the prediction drug repositioning on the association of new drugs and diseases. Compared with the simple average fusion method, DRDA can efficiently distinguish valid and invalid data sources, although it has a loss in precision. DRDA is thus performs better when predicting drug repositioning.

Although significant achievements have been made in drug repositioning technology due to research at home and abroad, evaluation indicators that describe drug repositioning accurately cannot be found in existing studies, despite being noted in the literature [24]. A drug repositioning algorithm should be evaluated with the combination of related information, such as predicted results and side effects, rather than directly considering the predicted new drug-disease result as erroneous. Thus, the performance of the drug repositioning algorithm cannot be accurately determined with indicators such as precision, recall rate, and AUC. Follow-up research should focus on enriching and improving existing evaluation indicators, and expanding data sources to achieve accurate disease similarity computation based on the current study.

## Supplementary Information

**Additional file 1**. **Fig. 8** an example of the similarity matrix obtained by the similarity calculation formula. **Fig. 9** an example of drug-disease association data.

## Data Availability

All the original data come from the literature [6] of Prof. Zhang. The processed datasets during the current study are available from the author on reasonable request.

## Abbreviations

AUC: The area under the receiver operating characteristic curve
DRDA: A Drug Repositioning Algorithm based on a Deep Autoencoder and Adaptive Fusion
DRCFFS: Computational drug repositioning using collaborative filtering by fusing multisource data
SLAMS: Similarity-based LArge-margin learning of multiple sources
DRBC: Drug repositioning algorithm based on collaborative filtering
UMLS: Unified Medical Language System
