# Supplementary material for: A drug repositioning algorithm based on a deep autoencoder and adaptive fusion
Source: BMC Bioinformatics. 2021 Oct 30;22:532. doi: 10.1186/s12859-021-04406-y (PMC8556784; doi:10.1186/s12859-021-04406-y)
Supplement: Supplementary file 1 — Additional file 1. Fig. 8 an example of the similarity matrix obtained by the similarity calculation formula. Fig. 9 an example of drug-disease association data. [file 12859_2021_4406_MOESM1_ESM.docx]

# Supplementary material


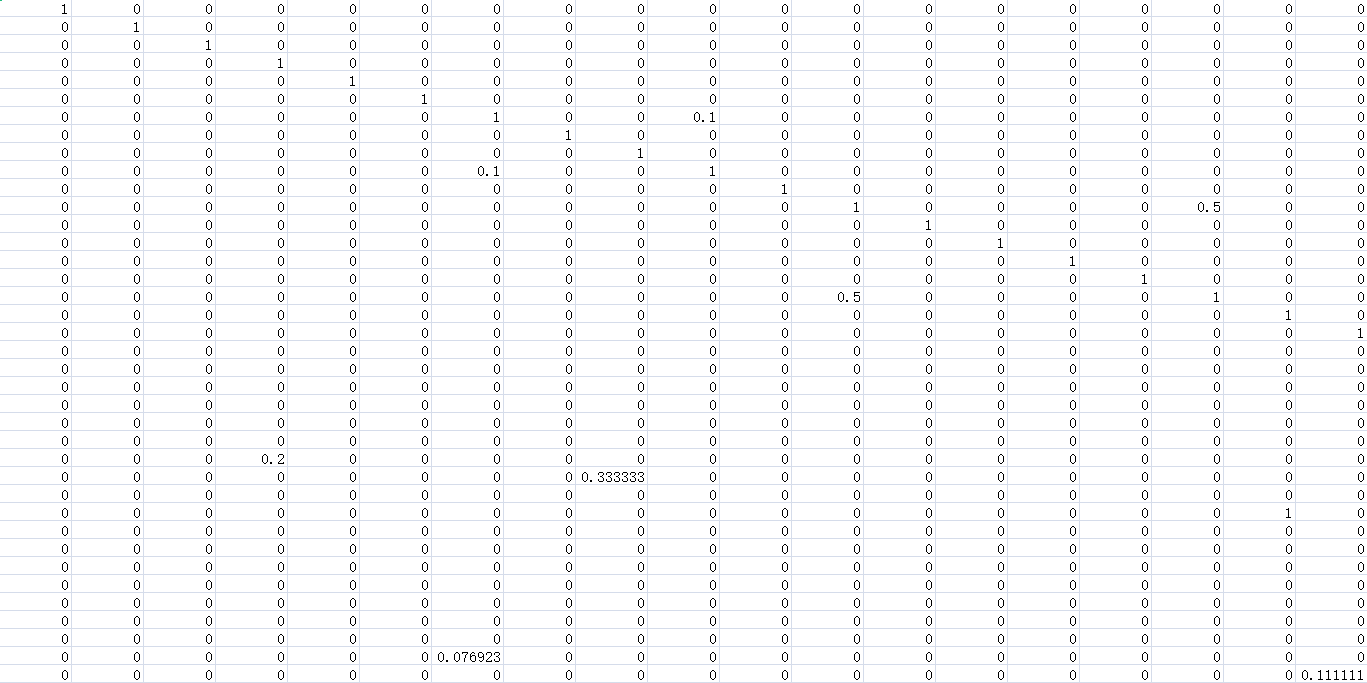


**Figure 8.** an example of the similarity matrix obtained by the similarity calculation formula


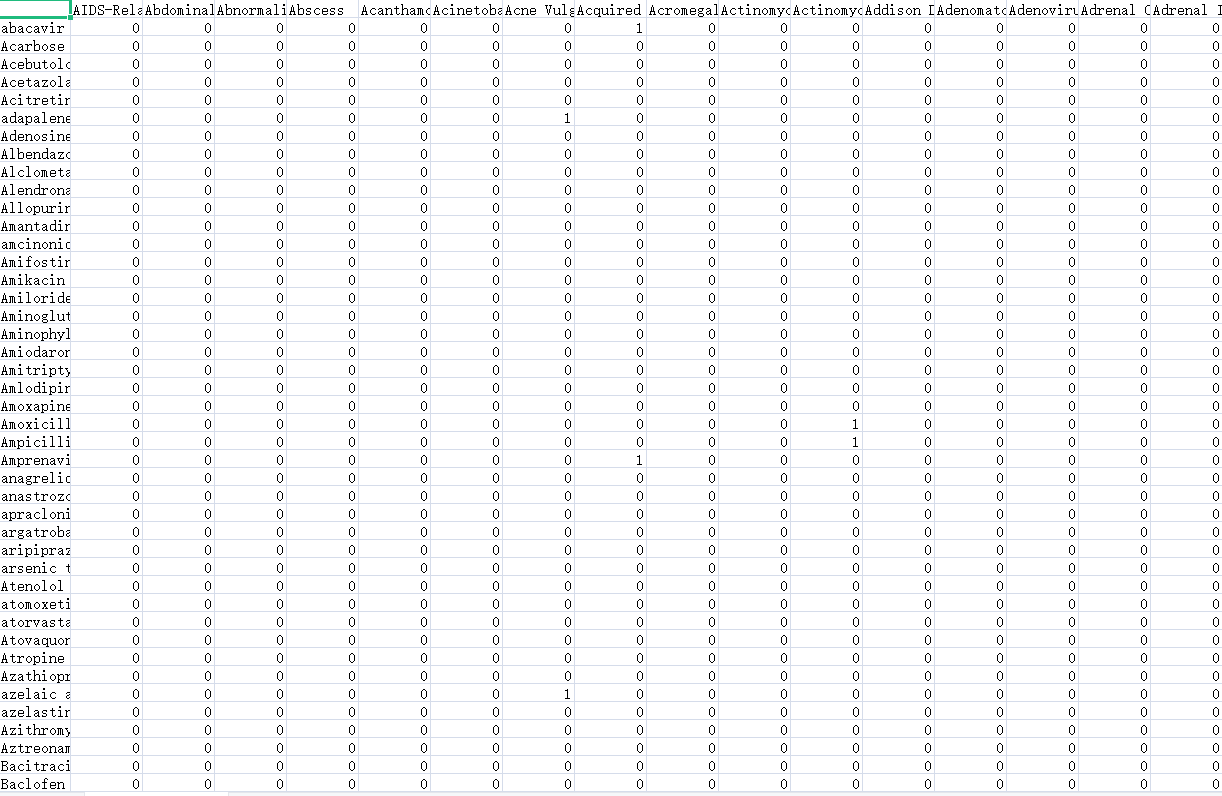


**Figure 9.** an example of drug-disease association data
